# GNSS Data/Pilot Combining with Extended Integrations for Carrier Tracking

**DOI:** 10.3390/s23083932

**Published:** 2023-04-12

**Authors:** Daniele Borio

**Affiliations:** European Commission, Joint Research Centre (JRC), 21027 Ispra, Italy; daniele.borio@ec.europa.eu

**Keywords:** bit estimation, GNSS signals, squaring, PLL, Phase Lock Loop, tracking

## Abstract

Modern Global Navigation Satellite System (GNSS) signals are usually made of two components: a pilot and a data channel. The former is adopted to extend the integration time and improve receiver sensitivity, whereas the latter is used for data dissemination. Combining the two channels allows one to fully exploit the transmitted power and further improve receiver performance. The presence of data symbols in the data channel, however, limits the integration time in the combining process. When a pure data channel is considered, the integration time can be extended using a squaring operation, which removes the data symbols without affecting phase information. In this paper, Maximum Likelihood (ML) estimation is used to derive the optimal data-pilot combining strategy and extend the integration time beyond the data symbol duration. In this way, a generalized correlator is obtained as the linear combination of the pilot and data components. The data component is multiplied by a non-linear term, which compensates for the presence of data bits. Under weak signal conditions, this multiplication leads to a form of squaring, which generalizes the squaring correlator used in data-only processing. The weights of the combination depend on the signal amplitude and noise variance that need to be estimated. The ML solution is integrated into a Phase Lock Loop (PLL) and used to process GNSS signals with data and pilot components. The proposed algorithm and its performance are characterized from a theoretical point of view, using semi-analytic simulations and through the processing of GNSS signals generated using a hardware simulator. The derived method is compared with other data/pilot combining strategies with extended integrations showing the advantages and drawbacks of the different approaches.

## 1. Introduction

Modern Global Navigation Satellite Systems (GNSSs) have brought several innovations from the signal design to the final user services, which are no longer limited to the user location and support, for instance, authentication and high-accuracy functions [1,2]. At the signal level, one of the main innovations brought by modern GNSSs has been the introduction of a pilot channel, which is now broadcast along with the traditional data channel used for navigation message dissemination [3]. Pilot signals have been adopted by the major GNSSs including GPS [3,4], Galileo [5,6] and BeiDou Navigation Satellite System (BDS) [7].

The absence of navigation bits on a pilot channel allows the coherent extension of the integration time and the adoption of a four-quadrant arctangent discriminator for carrier tracking [3,8]. These approaches, in turn, significantly extend the sensitivity of a GNSS receiver. A pilot component can also be used to support data channel processing and improve data demodulation performance [9].

The drawback of introducing a pilot channel is that the transmitted power is split between the data and pilot components [8]: if pilot-only processing is adopted, the signal energy on the data component is lost reducing the overall processing efficiency.

For this reason, since the early adoption of pilot signals, significant research has been conducted in order to develop efficient data/pilot combining strategies both at the acquisition [10,11] and at the tracking level [12,13,14,15].

With respect to carrier tracking, which is the main focus of this paper, several data/pilot combining strategies have been proposed and analyzed [12,13,16,17,18]. As discussed by [18], data/pilot combining can be implemented at three different levels in a tracking loop: (1) at the discriminator output, (2) at the correlator output, and (3) at the loop filter level. In all the approaches, data and pilot signals are correlated with local code and carrier replicas, and separate correlator outputs are obtained. In the first case, discriminators’ outputs are computed from the data and pilot correlators and combined to form new phase errors that are driven to zero by the tracking loop. This type of approach, which is often denoted as Optimal Linear Combination (OLC) Phase Lock Loop (PLL), was introduced by [12] and studied by several authors [8,14,16,17,18,19,20]. While simple and effective, it requires the estimation of the optimal weights used for the linear combination of the discriminator outputs [20]. These weights are a function of the variances of the discriminator outputs: while there exist theoretical formulas for computing these variances, non-linear effects at low Carrier-to-Noise power spectral density ratio ($C/N_{0}$) values make their evaluation deviate from theoretical results. In particular, it was recognized that under weak signal conditions the contribution of the data discriminator becomes unreliable and only the pilot component should be used. For this reason, adaptive approaches, where the weights are estimated from the input signals, were proposed [14,17]. At low $C/N_{0}$ the weight for the data contribution should tend to zero.

In the second case, the data and pilot correlators are combined to form a new joint correlator that should be used for the computation of the joint data/pilot discriminator. In particular, a non-linear function is used to estimate and remove the data bits from the data correlator that can then be coherently summed with the pilot component. The authors of [13] computed the joint log-likelihood associated with the data and pilot samples and used it as a residual error to be driven to zero in a joint data/pilot PLL. While the log-likelihood derived by [13] can be considered as a form of correlator combining strategy, it was used directly as discriminator output. For this reason, [18] considered it as a special case of the OLC PLL, where a hyperbolic tangent discriminator is adopted. Moreover, the approach suggested by [13] has some drawbacks that prevent its simple implementation. In particular, in [13] there are no considerations with respect to the discriminator gain: the derived log-likelihood depends on the input signal amplitude, and without proper normalization the loop proposed may become unstable. In this paper, the Maximum Likelihood (ML) approach from [13] is adopted and extended in order to compute joint data/pilot correlators.

Other forms of correlator-level combining are based on the decision-directed principle, where the data bits are estimated as the sign of the real part of the data correlator. A decision-directed PLL is obtained as an approximation of the ML approach of [13] for high $C/N_{0}$ values and has been considered for instance by [21].

The last approach, that is data/pilot combing at the loop filter output, has been considered by [22] and implies the use of two different loop filters for the data and pilot components.

Despite more than two decades of research on the topic of data/pilot combining, renewed interest in the subject has recently emerged due to the completion of BDS and the possibilities opened by the Beidou signals [19,20].

Moreover, when combining data and pilot signals, the integration time at the correlator level is often limited by the data bit duration [18]. While this issue is solved in the decision-directed approach, this strategy is not optimal at low $C/N_{0}$ values.

For these reasons, the joint problem of extended integrations and data/pilot optimum combining is considered in this paper. In particular, the ML estimation approach of [13] is used to derive the optimal data/pilot combining strategy and extend the integration time beyond the data symbol duration. In this way, a generalized correlator is obtained as the linear combination of the pilot and data components. The data component is multiplied by a non-linear term, which compensates for the presence of data bits. This correlator is denoted as Linear-Non-Linear (LNL) given the two types of contributions from the pilot and data components. Under weak signal conditions, this multiplication leads to a form of squaring, which generalizes the squaring correlator used in data-only processing [23]. The weights of the combination depend on the signal amplitude and noise variance that need to be estimated. Differently from [13], an equivalent correlator is found, and several data bit periods are considered. The ML solution is integrated into a PLL using a four-quadrant arctangent discriminator and used to process GNSS signals with data and pilot components. In this respect, the new correlator can be used as a pilot correlator and integrated into advanced algorithms for example for meta-signal processing [24,25].

The proposed algorithm and its performance are characterized from a theoretical point of view, using semi-analytic simulations and through the processing of GNSS signals generated using a hardware simulator. In particular, a Spirent GSS9000 simulator has been used to generate Galileo E1B/C signals with a progressively decreasing power. The signals have then been used to assess the performance of the proposed approach under different signal power conditions.

The derived method is compared with other data/pilot combining strategies with extended integrations showing the advantages and drawbacks of the different approaches.

The remainder of this paper is organized as follows: Section 2 introduces the signal and noise model. The LNL correlator is derived and analyzed in Section 3 along with the functional scheme of the related PLL. Section 4 theoretically analyses the LNL correlator loop performance. Simulation results and actual receiver processing are discussed in Section 5. Finally, conclusions are drawn in Section 6.

## 2. Signal and System Model

A GNSS signal made of both data and pilot components can be modeled at the receiver antenna as [3]:

(1) $$\begin{array}{cc} y(t) & =\sqrt{2C\alpha }d\left(t-\tau_{0}\right)c_{d}\left(t-\tau_{0}\right)\cos\left(2\pi \left(f_{RF}+f_{0}\right)t+\varphi_{0}+\varphi_{d}\right)+\\ \; & \sqrt{2C}c_{p}\left(t-\tau_{0}\right)\cos\left(2\pi \left(f_{RF}+f_{0}\right)t+\varphi_{0}\right)+\eta \left(t\right), \end{array}$$

that is the sum of three terms: the data and the pilot components and an Additive White Gaussian Noise (AWGN), η(t). In (1), *C* is the received power of the pilot signal, whereas α is the ratio between the data and pilot power levels. Differences in power levels may be due to signal design choices. For instance, α=1 for the Galileo E1B/C signals [6], whereas in the Beidou B1 case, α=0.25 [7].

d(·) is the navigation message whereas $c_{d}\left(\cdot \right)$ and $c_{p}\left(\cdot \right)$ denote the pseudorandom codes, including the effect of secondary codes, of the data and pilot components, respectively. $\tau_{0}$, $f_{0}$ and $\varphi_{0}$ are the delays, Doppler frequency, and carrier phase introduced by the communication channel. The effect of the Doppler shift on the code components is neglected. *t* is the time variable and $f_{RF}$ is the signal Radio Frequency (RF). The data and pilot components can be transmitted in different phases. This effect is modeled through $\varphi_{d}$, which is known. In the Galileo E1B/C case, the two components are broadcast in opposition of phase and $\varphi_{d}=\pi$.

While several useful signals are received at the same time from different satellites, a GNSS receiver is able to process them separately exploiting the correlation properties of the pseudorandom codes. For this reason, a single useful signal is considered in (1).

Signal (1) is filtered, downconverted to baseband, and digitized. In this way, the following digital signal model is obtained:

(2) $$\begin{array}{cc} y_{bb}\left[n\right]=y_{bb}\left(nT_{s}\right) & =\sqrt{C\alpha }e^{j\varphi_{d}}d\left(nT_{s}-\tau_{0}\right)c_{d}\left(nT_{s}-\tau_{0}\right)e^{j2\pi f_{0}nT_{s}+j\varphi_{0}}\\ \; & +\sqrt{C}c_{p}\left(nT_{s}-\tau_{0}\right)e^{j2\pi f_{0}nT_{s}+j\varphi_{0}}+\eta_{bb}\left(nT_{s}\right), \end{array}$$

where subscript, bb, indicates quantities brought to baseband. *n* is the time index and $T_{s}$ is the sampling interval. The inverse of $T_{s}$ is the sampling frequency, $f_{s}$. $\eta_{bb}\left(t\right)$ is zero mean with independent and identically distributed (i.i.d.) real and imaginary parts, both with variance $\sigma_{\eta }^{2}$. This variance depends on several factors related to the antenna and front-end implementation. A commonly adopted model for $\sigma_{\eta }^{2}$ is

(3) $$\sigma_{\eta }^{2}=N_{0}B_{Rx},$$

where $B_{Rx}$ is the front-end one-sided bandwidth and $N_{0}$ is the Power Spectral Density (PSD) of the input noise, η(t). The ratio between the carrier power, *C*, and the noise PSD, $N_{0}$, defines the $C/N_{0}$ that is continuously estimated by the receiver.

In standard receiver processing, $y_{bb}\left[n\right]$ is correlated with local replicas of the carrier and the data and pilot codes. *N* samples are used for the computation of each correlator and $T_{c}=NT_{s}$, the coherent integration time, is limited by the bit duration in the data navigation message, d(·). The same coherent integration time is assumed for both components. Several correlators are computed using subsequent portions of the input signal, (2). In this way, a set of correlators is obtained. If the effect of the residual Doppler frequency is neglected, the following model can be adopted for the data and pilot correlators:

(4) $$P_{p,i}=AR_{p}\left(\Delta \tau \right)e^{j\Delta \varphi }+\eta_{p,i}$$

(5) $$P_{d,i}=kAd_{i}R_{d}\left(\Delta \tau \right)e^{j\Delta \varphi }+\eta_{d,i}$$

where *i* is the temporal index used to indicate correlators computed from the different signal portions. $\Delta \tau =\tau -\tau_{0}$ is the residual delay error and τ is the delay tested by the receiver. Similarly, $\Delta \varphi =\varphi -\varphi_{0}$ is the residual carrier phase with φ the phase tested by the receiver. $d_{i}=d\left[iNT_{s}\right]$ is the symbol transmitted by the data navigation message and assumed constant over the coherent integration time. $A=\sqrt{C}$ is the signal amplitude and $k=\sqrt{\alpha }e^{j\varphi_{d}}$ is a known constant taking into account possible amplitude/phase differences between the data and pilot components.

In (4) and (5), $R_{d}\left(\cdot \right)$ and $R_{p}\left(\cdot \right)$ are the data and pilot correlation functions, respectively. The different noise components, $\eta_{p,i}$ and $\eta_{d,i}$ are zero-mean i.i.d. Gaussian random variables. The real and imaginary parts of $\eta_{p,i}$ and $\eta_{d,i}$ have each variance, $\sigma^{2}=\frac{\sigma_{\eta }^{2}}{N}$, where *N* is the number of samples used in the correlation process.

The focus of this paper is the optimal, in the ML sense, estimation of the phase Δφ using data and pilot correlators from several epochs. For this reason, it is assumed that the Delay Lock Loop (DLL) is able to properly recover the signal delay and Δτ≈0 and $R_{d}\left(\Delta \tau =0\right)=1$ and $R_{p}\left(\Delta \tau =0\right)=1$.

## 3. Linear-Non-Linear Correlator

Using the signal model introduced in the previous section, it is possible to show (see Appendix A) that the ML estimate, $\Delta \hat{\varphi }$, of Δφ is given by the solution of the following equation:

(6) $$\Delta \hat{\varphi }=∠\left[\sum_{i=0}^{K-1}P_{p,i}+\sum_{i=0}^{K-1}\tanh\left(\frac{A}{\sigma^{2}}ℜ\left\{{\tilde{P}}_{d,i}e^{-j\Delta \hat{\varphi }}\right\}\right){\tilde{P}}_{d,i}\right].$$

As defined in Appendix A, ${\tilde{P}}_{d,i}=k^{*}\cdot P_{d,i}$ are the data correlators corrected for the known factor *k*. Symbol ${\left(.\right)}^{*}$ denotes complex conjugation.

The term between square brackets in (6) defines a generalized correlator where the integration time is extended coherently on the pilot component (linear part) and non-linearly on the data correlator. This correlator, denoted as the LNL correlator, is given by:

(7) $$P_{ln}=\sum_{i=0}^{K-1}P_{p,i}+\sum_{i=0}^{K-1}\tanh\left(\frac{A}{\sigma^{2}}ℜ\left\{{\tilde{P}}_{d,i}e^{-j\Delta \hat{\varphi }}\right\}\right){\tilde{P}}_{d,i}.$$

The computation of (7) requires the knowledge of $\Delta \hat{\varphi }$, which appears on both sides of (6). However, under phase lock conditions it is possible to assume:

(8) $$\Delta \hat{\varphi }\approx 0\;and\;e^{-j\Delta \hat{\varphi }}\approx 1.$$

The effectiveness and validity of this assumption have been verified through simulations as better discussed in the following sections.

Using (8), the final LNL is obtained as:

(9) $$P_{ln}=\sum_{i=0}^{K-1}P_{p,i}+\sum_{i=0}^{K-1}\tanh\left(\frac{A}{\sigma^{2}}ℜ\left\{{\tilde{P}}_{d,i}\right\}\right){\tilde{P}}_{d,i}.$$

**Figure 1:**
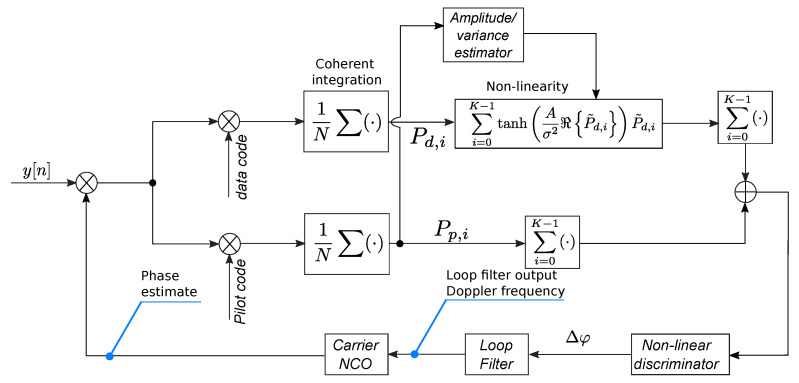
Schematic representation of a PLL integrating the LNL correlator and fully exploiting the availability of data and pilot components.

Once computed, the LNL correlator can be used as a pure pilot correlator in a standard pilot PLL architecture. The integration of the LNL correlator within a PLL architecture is shown in Figure 1. The data and pilot signals are integrated on two different processing branches and the data and pilot correlators are computed. The pilot correlators are fed to a simple amplitude and variance estimator block, which provides estimates for *A* and $\sigma^{2}$, which in turn are used for the computation of (7). Data and pilot correlators are combined in a non-linear way and the LNL correlator is obtained. Finally, $P_{ln}$ is passed through a standard four-quadrant arctangent discriminator [3] and the following discriminator output is obtained:

(10) $$\Delta \varphi_{d}=∠P_{ln}=\arctan_{2}\left(ℑ\left\{P_{ln}\right\},ℜ\left\{P_{ln}\right\}\right).$$

The residual phase error, $\Delta \varphi_{d}$, passes through the remaining parts of the loop, which are standard and include a loop filter and a carrier Numerically Controlled Oscillator (NCO) [3]. The NCO generates the local carrier that is used for the correlation process, closing the loop.

### 3.1. Weak Signal Conditions

Under weak signal conditions, i.e., for low $C/N_{0}$ values, the argument of the hyperbolic tangent in (9) approaches zero. Under these conditions, the hyperbolic tangent can be approximated as

tanh(x)≈x

and the LNL correlator can be expressed as:

(11) $$\begin{array}{cc} P_{ln} & \approx \sum_{i=0}^{K-1}P_{p,i}+\frac{A}{\sigma^{2}}\sum_{i=0}^{K-1}ℜ\left\{{\tilde{P}}_{d,i}\right\}{\tilde{P}}_{d,i}\\ \; & =\sum_{i=0}^{K-1}P_{p,i}+\frac{A}{2\sigma^{2}}\sum_{i=0}^{K-1}\left[{\tilde{P}}_{d,i}^{2}+{\left|{\tilde{P}}_{d,i}\right|}^{2}\right] \end{array}$$

that is the weighted sum of pilot correlators, with extended coherent integrations, and the data correlators, which are multiplied by the real part. When $ℜ\left\{{\tilde{P}}_{d,i}\right\}$ is expressed as the average of the data correlators and their complex conjugates, squaring operations are clearly highlighted: in this way, the square of data correlators and their squared modulus are found. In this respect, the LNL correlator can be interpreted as a generalization of the squaring correlator [23] effectively combining both data and pilot components. Finally, when very weak signal conditions occur, i.e for $\frac{A^{2}}{\sigma^{2}}\ll 1$, the only surviving term in (11) is the one related to the pilot correlators and

(12) $$P_{ln}\approx \sum_{i=0}^{K-1}P_{p,i}.$$

Thus, the LNL correlator degenerates to a pure pilot correlator with extended coherent integration time. This result is consistent with previous findings from the literature [14,18] that indicate that data information should be discarded under weak signal conditions.

### 3.2. Strong Signal Conditions

Under strong signal conditions, i.e., for $\frac{A^{2}}{\sigma^{2}}\gg 1$, the hyperbolic tangent can be replaced by the sign of its argument [13] and

(13) $$P_{ln}\approx \sum_{i=0}^{K-1}P_{p,i}+\sum_{i=0}^{K-1}\mathrm{sign}\left(ℜ\left\{{\tilde{P}}_{d,i}\right\}\right){\tilde{P}}_{d,i}$$

where the ‘sign(.)’ function is equal to 1 if its argument is positive and −1 if negative. Equation (13) is the decision-directed correlator considered for instance in [19,21].

## 4. Tracking Jitter

The tracking jitter determines the amount of noise transferred from the input signal, y[n], to the final phase estimate used by the NCO to generate the local carrier. The tracking jitter is defined as [26]:

(14) $$\sigma_{\varphi }=\frac{\sigma_{d}}{G_{s}}\sqrt{2B_{eq}T_{u}}$$

where $\sigma_{\varphi }$ is the tracking jitter and $\sigma_{d}$ is the standard deviation of the discriminator output (10). $G_{d}$ is the discriminator gain, $B_{eq}$ is the loop equivalent bandwidth and $T_{u}$ is the loop update rate equal to $KT_{c}$. The normalized bandwidth, $B_{eq}T_{u}$, is used to propagate the standard deviation from the discriminator output to the NCO output.

The discriminator gain is defined as [26]

(15) $$G_{d}={\left.\frac{\partial E\left[\Delta \varphi_{d}\right]}{\partial \Delta \varphi }\right|}_{\Delta \varphi =0}={\left.\frac{\partial S(\Delta \varphi )}{\partial \varphi }\right|}_{\Delta \varphi =0},$$

where S(Δφ) is the discriminator input-output function expressed as a function of the input error, Δφ.

For arctangent discriminators as (10), it is shown that [27]:

(16) $$\frac{\sigma_{d}}{G_{d}}=\sqrt{\frac{R_{s}+1}{R_{s}^{2}}}$$

where $R_{s}$ is the Signal-to-Noise Ratio (SNR) at the input of the arctangent discriminator:

(17) $$R_{s}=\frac{{\left|E\left\{P_{ln}\right\}\right|}^{2}}{\frac{1}{2}Var\left\{P_{ln}\right\}}.$$

$E\left\{P_{ln}\right\}$ and $\mathrm{Var}\left\{P_{ln}\right\}$ can be approximately determined using an approach similar to that adopted by [13]. In particular,

(18) $$\begin{array}{cc} E\left\{P_{ln}\right\} & =E\left\{\sum_{i=0}^{K-1}P_{p,i}+\sum_{i=0}^{K-1}\tanh\left(\frac{A}{\sigma^{2}}ℜ\left\{{\tilde{P}}_{d,i}\right\}\right){\tilde{P}}_{d,i}\right\}\\ \; & =\sum_{i=0}^{K-1}E\left\{P_{p,i}\right\}+\sum_{i=0}^{K-1}E\left\{\tanh\left(\frac{A}{\sigma^{2}}ℜ\left\{{\tilde{P}}_{d,i}\right\}\right){\tilde{P}}_{d,i}\right\}\\ \; & =K\left[Ae^{j\Delta \varphi }+E\left\{\tanh\left(\frac{A}{\sigma^{2}}ℜ\left\{{\tilde{P}}_{d,i}\right\}\right){\tilde{P}}_{d,i}\right\}\right]. \end{array}$$

Equation (18) can be simplified by assuming that phase lock conditions, i.e., Δφ≈0. Moreover, the delta method [28] (Appendix A) can be used to approximate the remaining expected value in the last line of (18):

(19) $$\begin{array}{cc} E\left\{\tanh\left(\frac{A}{\sigma^{2}}ℜ\left\{{\tilde{P}}_{d,i}\right\}\right){\tilde{P}}_{d,i}\right\} & \approx \tanh\left(\frac{A}{\sigma^{2}}E\left\{ℜ\left\{{\tilde{P}}_{d,i}\right\}\right\}\right)E\left\{{\tilde{P}}_{d,i}\right\}\\ \; & \approx \tanh\left(\frac{A^{2}{\left|k\right|}^{2}}{\sigma^{2}}\right)A{\left|k\right|}^{2}. \end{array}$$

In this way, (18) becomes:

(20) $$E\left\{P_{ln}\right\}\approx KA\left[1+{\left|k\right|}^{2}\tanh\left(\frac{A^{2}{\left|k\right|}^{2}}{\sigma^{2}}\right)\right].$$

Similarly, it is possible to evaluate an approximation for $\mathrm{Var}\left\{P_{ln}\right\}$:

(21) $$\begin{array}{cc} \mathrm{Var}\left\{P_{ln}\right\} & =\mathrm{Var}\left\{\sum_{i=0}^{K-1}P_{p,i}+\sum_{i=0}^{K-1}\tanh\left(\frac{A}{\sigma^{2}}ℜ\left\{{\tilde{P}}_{d,i}\right\}\right){\tilde{P}}_{d,i}\right\}\\ \; & =K\left[\mathrm{Var}\left\{P_{p,i}\right\}+\mathrm{Var}\left\{\tanh\left(\frac{A}{\sigma^{2}}ℜ\left\{{\tilde{P}}_{d,i}\right\}\right){\tilde{P}}_{d,i}\right\}\right]\\ \; & \approx K\left[2\sigma^{2}+\mathrm{Var}\left\{\tanh\left(\frac{A}{\sigma^{2}}E\left\{ℜ\left\{{\tilde{P}}_{d,i}\right\}\right\}\right){\tilde{P}}_{d,i}\right\}\right]\\ \; & =2K\sigma^{2}\left[1+{\left|k\right|}^{2}\tanh^{2}\left(\frac{A^{2}{\left|k\right|}^{2}}{\sigma^{2}}\right)\right]. \end{array}$$

Using these results, it is finally possible to compute $R_{s}$:

(22) $$R_{s}\approx K\frac{A^{2}}{\sigma^{2}}\frac{{\left[1+{\left|k\right|}^{2}\tanh\left(\frac{A^{2}{\left|k\right|}^{2}}{\sigma^{2}}\right)\right]}^{2}}{1+{\left|k\right|}^{2}\tanh^{2}\left(\frac{A^{2}{\left|k\right|}^{2}}{\sigma^{2}}\right)}.$$

Using the model introduced in Section 2, it is possible to express the SNR at the correlator output as a function of the coherent integration time, $T_{c}$, and the $C/N_{0}$. In particular,

(23) $$\frac{A^{2}}{\sigma^{2}}=2\frac{C}{N_{0}}T_{c}$$

and

(24) $$R_{s}\approx 2K\frac{C}{N_{0}}T_{c}\frac{{\left[1+{\left|k\right|}^{2}\tanh\left(2\frac{C}{N_{0}}{\left|k\right|}^{2}T_{c}\right)\right]}^{2}}{1+{\left|k\right|}^{2}\tanh^{2}\left(2\frac{C}{N_{0}}{\left|k\right|}^{2}T_{c}\right)}.$$

Finally, tracking jitter (14) assumes the following form:

(25) $$\sigma_{\varphi }=\sqrt{\frac{B_{eq}}{C/N_{0}}\frac{1+{\left|k\right|}^{2}\tanh^{2}\left(2\frac{C}{N_{0}}{\left|k\right|}^{2}T_{c}\right)}{{\left[1+{\left|k\right|}^{2}\tanh\left(2\frac{C}{N_{0}}{\left|k\right|}^{2}T_{c}\right)\right]}^{2}}\left(1+\frac{1}{2K\frac{C}{N_{0}}T_{c}}\frac{1+{\left|k\right|}^{2}\tanh^{2}\left(2\frac{C}{N_{0}}{\left|k\right|}^{2}T_{c}\right)}{{\left[1+{\left|k\right|}^{2}\tanh\left(2\frac{C}{N_{0}}{\left|k\right|}^{2}T_{c}\right)\right]}^{2}}\right)}.$$

## 5. Results and Discussion

The performance of a PLL implementing the LNL correlator is analyzed in the following using semi-analytic simulations and actual receiver processing, implemented using a Software Defined Radio (SDR) approach.

### 5.1. Semi-Analytic Simulation Results

In order to support the theoretical results presented in the previous section, semi-analytic simulations have been performed considering different loop parameters. Sami-analytic simulations leverage the analytical knowledge of a system to reduce the computation load and time that full Monte Carlo simulations would require [29]. In this case, we use an analytical model to describe the correlation process, which is the most computationally intensive element in GNSS receiver processing. All the other elements of the loop are simulated. In particular, we used the semi-analytic framework developed in [30] and extended it to process both data and pilot components, which are then combined to form the LNL correlator introduced in Section 3.

For the simulations, the parameters reported in Table 1 were used: for all the tests, a sampling frequency, $f_{s}=4$ MHz was used. Moreover, for each $C/N_{0}$ value, $5\times 10^{5}$ simulation runs were performed and for each run a phase error was found. These simulated phase errors were used for the computation of the tracking jitter. Details on the semi-analytic procedure adopted can be found in [30].

For all the simulation experiments, a data/pilot power ratio, α=1, was assumed. Different coherent integration times, loop bandwidths, and orders were considered.

**Figure 2:**
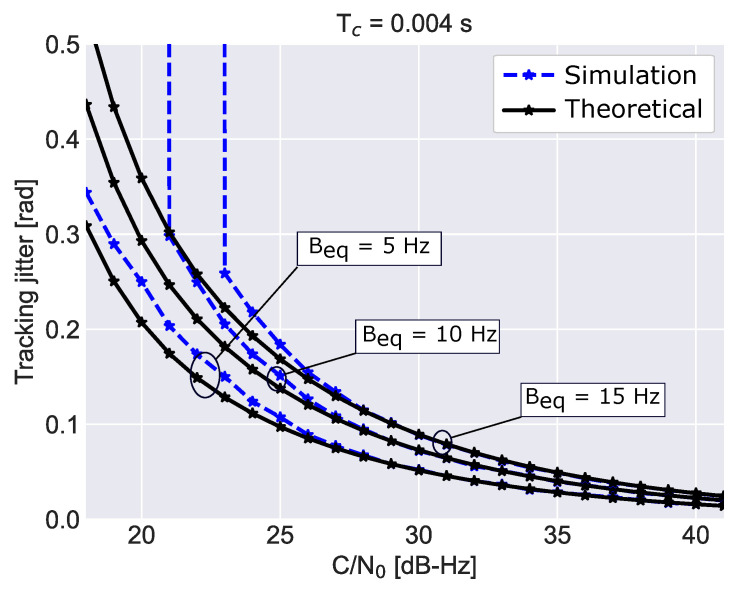
Comparison between the theoretical tracking jitter and simulation results. Different loop equivalent bandwidths are considered for a single non-coherent integration, K=1.

The case for K=1 is studied at first. In particular, the effect of different loop bandwidths on the tracking jitter is studied in Figure 2 for a fixed coherent integration time, $T_{c}=4$ ms, for a single non-coherent integration, K=1, and for a third order loop. As expected the tracking jitter decreases with the loop bandwidth. A good agreement is found between theoretical and simulation results. Differences are found for $C/N_{0}$ lower than 27 dB-Hz. The vertical trends visible for the curves obtained for equivalent bandwidths equal to 10 and 15 Hz are due to non-linear phenomena occurring at low $C/N_{0}$ values. Under weak signal conditions, the loop loses its lock and the tracking jitter diverges. When the loop bandwidth is decreased, loss of lock occurs at lower $C/N_{0}$ values. Loss of lock effects are not accounted for in the theoretical tracking jitter derived in the previous section, which is based on the linear theory of tracking loops.

**Figure 3:**
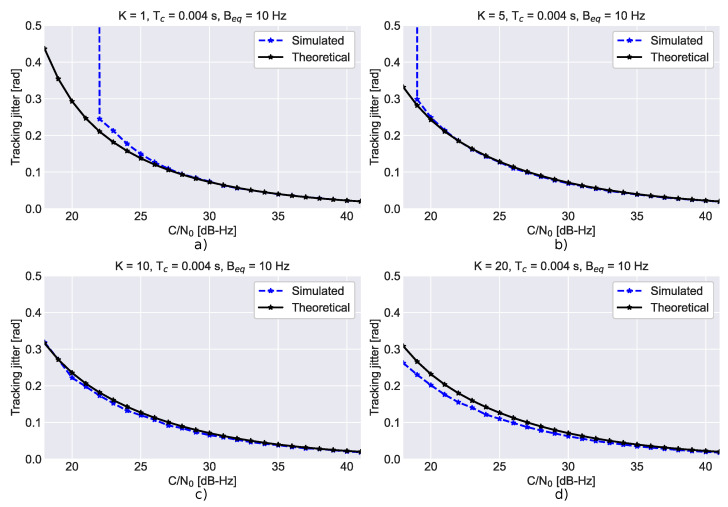
Comparison between the theoretical tracking jitters and simulation results for different non-coherent integrations. (**a**) K=1, (**b**) K=5, (**c**) K=10, (**d**) K=20.

The impact of the number of non-coherent integrations is investigated in Figure 3, which compares the theoretical tracking jitters with simulation results for different numbers of non-coherent integrations, *K*. In order to improve the clarity of the figure, four subplots are provided. A third-order loop with a 10 Hz bandwidth and 4 ms coherent integration was considered. In all cases, a good agreement between theoretical and simulation results is found. Note that when the number of non-coherent integrations is increased better performance is achieved. The loop is more stable, lower differences between theoretical and simulation results are found and loss of lock occurs for lower $C/N_{0}$ values. This effect is due to the noise reduction caused by the non-coherent integration process.

### 5.2. Comparison with Other Data/Pilot Combining Strategies

In this section, the LNL correlator approach has been compared with other data/pilot combining techniques with extended integrations. In particular, the following approaches have been considered in addition to the LNL correlator:

- the decision-directed PLL obtained in (13), where data bits are removed through the sign operator,
- the OLC PLL where the data and pilot discriminator outputs are combined assigning the same weight to both components,
- pure pilot processing which is used as a baseline. Note that in this case, the integration time is extended coherently. Thus, the total coherent integration is given by $KT_{c}$.

**Figure 4:**
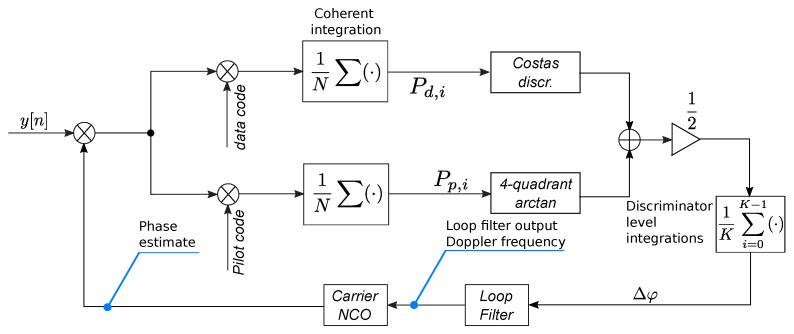
Schematic representation of the OLC PLL integrating extended integrations at the discriminator level.

It is important to note that extended integration times are not foreseen by the original OLC approach [12], whose integration time is limited by the duration of the data bits. In this paper, the integration time has been extended by averaging the discriminator outputs for *K* epochs. This approach has been adopted to perform a fair comparison between the algorithms listed above. This principle is shown in Figure 4, which provides a schematic representation of the OLC PLL integrating extended integrations at the discriminator level. The data and pilot components are correlated separately and different discriminator outputs are computed. A Costas discriminator [3] is adopted for the data component whereas a four-quadrant arctangent discriminator is used for the pilot branch. The two discriminator outputs are combined according to the OLC principle and further integrated for *K* epochs. In this way, the OLC PLL has the same total integration time and update rate as the other approaches considered for the comparison.

**Figure 5:**
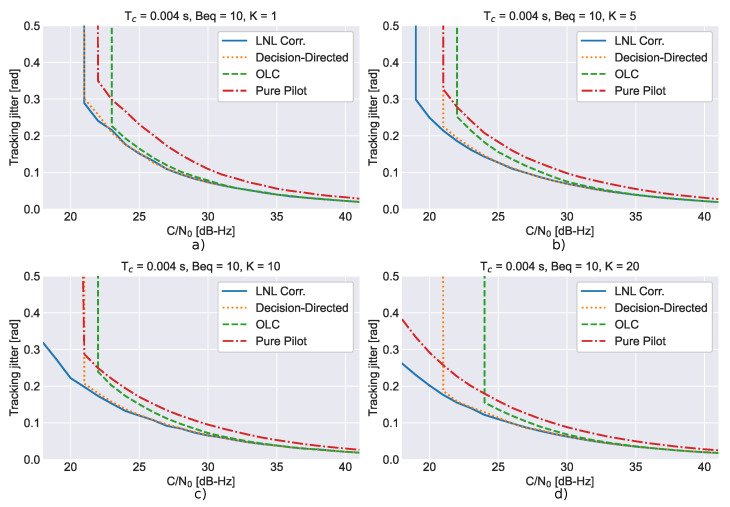
Comparison between the tracking jitters obtained using semi-analytic simulations for different data/pilot combining strategies with extended integration time. (**a**) K=1, (**b**) K=5, (**c**) K=10, (**d**) K=20.

The semi-analytic simulations described in Section 5.1 have been used to determine the tracking jitter of the different algorithms. The obtained tracking jitters are compared in Figure 5 for different integration times. In all cases, a coherent integration time equal to 4 ms has been adopted along with an equivalent loop bandwidth equal to 10 Hz. Third-order PLLs were considered. From the figure, it emerges that all data/pilot combing techniques have similar tracking jitters at high $C/N_{0}$ values. This is expected since, under good signal conditions, the so-called squaring loss [13], that is the loss caused by the non-linear effects of the discriminator function, becomes negligible and the different tracking jitter converges to the same performance. Pure pilot tracking, when signal combining is not implemented, suffers a loss of 3 dB with respect to the other techniques: this is also expected since the power of the data component is not recovered.

At low $C/N_{0}$ values, non-linear effects start impacting the performance of the different algorithms. The OLC PLL is the approach where loss of lock occurs first, that is for higher $C/N_{0}$ values as compared to the other PLLs. Moreover, the OLC PLL tracking jitter suffers degradations with respect to the other approaches. These degradations are more evident when the number of extended integrations is increased.

The decision-directed PLL defined by (13) always loses lock at the same $C/N_{0}$ level, around 21 dB-Hz, independently from the number of extended integrations. At such a low $C/N_{0}$ value, the bit estimation process performed using the sign function becomes unreliable and data/pilot combing becomes ineffective. The bit estimation process is independent of the number of extended integrations and this may explain the fact that the decision-directed PLL always loses lock at the same $C/N_{0}$ level. For $C/N_{0}$ values above 21 dB-Hz, the decision-directed PLL achieves a tracking jitter coinciding with that of the PLL implementing the LNL correlator: this confirms the fact that the decision-directed PLL approximates the LNL correlator for high $C/N_{0}$ values.

The LNL correlator and pure pilot tracking effectively benefit from the increased number of extended integrations, *K*. As *K* is increased, loss of lock occurs at progressively lower $C/N_{0}$ values. Overall the LNL correlator PLL is the algorithm achieving the best performance in terms of low tracking jitter and loss of lock under weak signal conditions.

### 5.3. Experimental Setup and Software Receiver Processing

**Figure 6:**
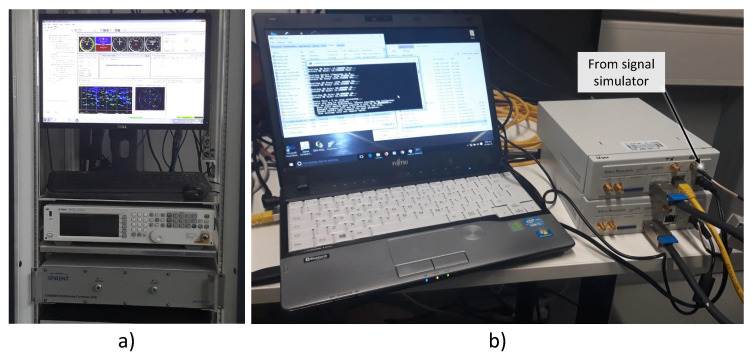
Experimental setup adopted for the collection of the I/Q samples generated using a Spirent GSS9000 simulator. (**a**) View of the Spirent GSS9000 simulator. (**b**) View of the USRP II adopted for signal recording.

Theoretical results and semi-analytic simulations have been complemented by experiments involving an SDR receiver implementing the LNL correlator for joint data/pilot tracking. More specifically, the experimental setup illustrated in Figure 6 has been adopted. A hardware Spirent GSS9000 simulator was used to simulate Galileo E1B/C signals with a progressively decreasing power. In particular, the power of all the satellite signals generated using the Spirent GSS9000 simulator has been progressively decreased by 1 dB each minute. A view of the Spirent GSS9000 simulator is provided in the left part of Figure 6.

In-phase Quadrature (I/Q) baseband data has been collected using a Universal Software Radio Peripheral (USRP) II platform and stored on disk for further processing. The parameters used for the collection of the I/Q data are reported in Table 2. In particular, a sampling frequency of 12.5 MHz was adopted along with 8 bits for signal quantization. A view of the USRP II used for signal recording is provided in Figure 6. The full experiment lasted about 25 min (1500 s) leading to Galileo signals characterized by $C/N_{0}$ values below 20 dB-Hz.

The data collected has been processed using a custom Python software receiver implementing LNL correlator tracking. The parameters used for tracking the Galileo E1 signals are reported in Table 3.

Note that the software receiver implements a standard processing architecture and only limited modifications have been introduced to support LNL correlation processing. A standard acquisition block, based on pilot-only processing, has been implemented along with a multi-state tracking architecture.

In the test conducted, ten Galileo satellite signals are present. These signals are correctly acquired by the receiver at the beginning of the test and further processed by the tracking block. Signals are processed in parallel by the receiver with negligible Multiple Access Interference (MAI). This is due to the fact that Galileo signals have been designed to minimize mutual cross-correlations and the received signals have similar power levels.

When moving to tracking, the receiver first uses a standard Frequency Lock Loop (FLL) to obtain frequency lock. Once achieved, processing switches to a standard Costas PLL until secondary code synchronization on the pilot channel is achieved. After recovering the secondary code, either pilot-only or combined data/pilot processing can finally start.

Pure pilot processing has been implemented for comparison purposes along with the decision-directed and OLC PLLs. In all cases considered, the data prompt correlator needs to be computed for demodulating the navigation message. Thus, the data prompt correlator is readily available and can be directly used for data/pilot combining.

Since, in this paper, we focused on combined data/pilot processing for phase tracking only, we adopted a standard DLL using only the pilot early and late correlators. While the LNL correlator can be used also for improving the DLL performance, this type of processing is out of the scope of this paper. Moreover, by modifying only the PLL, no additional correlators were needed for the evaluation of the LNL correlator with respect to pure pilot processing.

This shows that phase tracking using the LNL can be implemented with limited additional computational resources with respect to standard receiver architectures.

Finally, the computation of the LNL correlator, (9), requires the estimation of the correlator amplitude, *A*, and variance, $\sigma^{2}$. This has been achieved in the software receiver using a simple exponential filter on the pilot correlator. After achieving phase lock, the only noise is present on the quadrature component of the pilot prompt correlator, which is a zero mean. Thus, the noise variance is estimated as:

(26) $${\hat{\sigma }}_{i}^{2}=\gamma {\hat{\sigma }}_{i-1}^{2}+\left(1-\gamma \right)ℑ{\left\{P_{p,i}\right\}}^{2},$$

where ${\hat{\sigma }}_{i}^{2}$ is the estimated noise variance at the *i*th epoch. γ is the filter forgetting factor and is set to 0.99. $P_{p,i}$ is the pilot prompt correlator at epoch *i*. The first estimate of the noise variance is set equal to $ℑ{\left\{P_{p,0}\right\}}^{2}$, where, in this case, time index 0 indicates the first epoch when secondary code synchronization has been achieved.

The signal amplitude, *A*, can be estimated in a similar way using the real part of the pilot prompt correlator. After secondary code synchronization, the pilot prompt correlator assumes only positive values and *A* can be estimated as:

(27) $${\hat{A}}_{i}=\gamma {\hat{A}}_{i-1}+\left(1-\gamma \right)ℜ\left\{P_{p,i}\right\},$$

where ${\hat{A}}_{i}$ is the signal amplitude estimated at the instant *i*. Also, in this case, γ=0.99 was adopted.

In the next section, experimental results will also show the effectiveness of (26) and (27) in estimating the correlator amplitude and variance.

### 5.4. Experimental Results

Sample results, obtained processing the I/Q data collected according to the experimental setup described in the previous section, are presented in the following.

**Figure 7:**
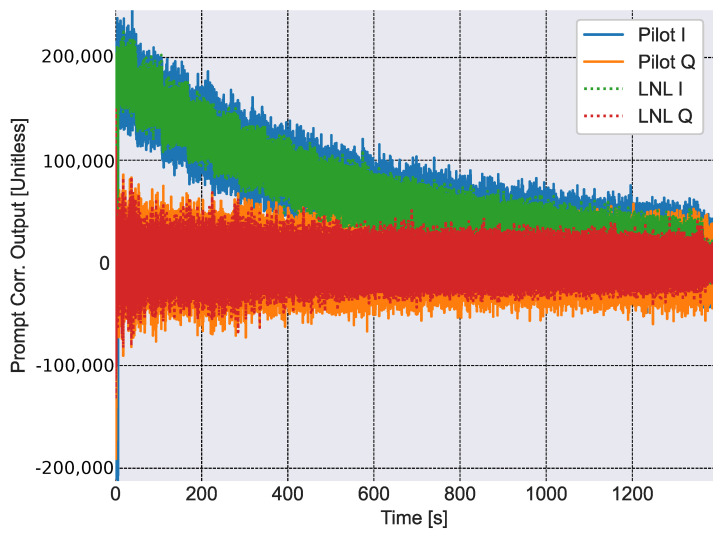
Comparison between the pure pilot and LNL correlators obtained for the different epochs of the experiment. A single non-coherent integration is considered.

At first, Figure 7 provides a comparison between the correlators obtained using pure pilot processing and the combined approach derived in this paper. A single integration, performed on 4 ms, is considered. In both cases, the receiver is able to properly recover the phase of the received signal and aligns all the useful energy in the in-phase branch of the correlators. The effect of the progressive signal attenuation, introduced in the simulated scenario, is also clearly visible on the in-phase components of the correlators whose amplitudes are progressively reduced. As expected, only positive values are observed in the in-phase correlator components after secondary code recovery. Loss of lock is observed in both cases after about 1400 s from the start of the test. Note that correlator outputs are obtained by processing the digital samples provided by the receiver front-end. Since these digital samples, whose values depend on the amplification and quantization strategy implemented by the receiver front-end, are unitless, also correlator outputs do not have a unit of measurement.

From the figure, the advantages of the LNL correlator, which has been scaled by a factor of 1/2 for comparison purposes, clearly emerge with respect to pure pilot processing: while the pilot and LNL correlators assume the same mean value and overlap, the latter is less noisy and are affected by lower noise levels. This is the advantage of combining together both data and pilot correlators: coherent combining is obtained through the multiplication by the hyperbolic tangent term in (9).

**Figure 8:**
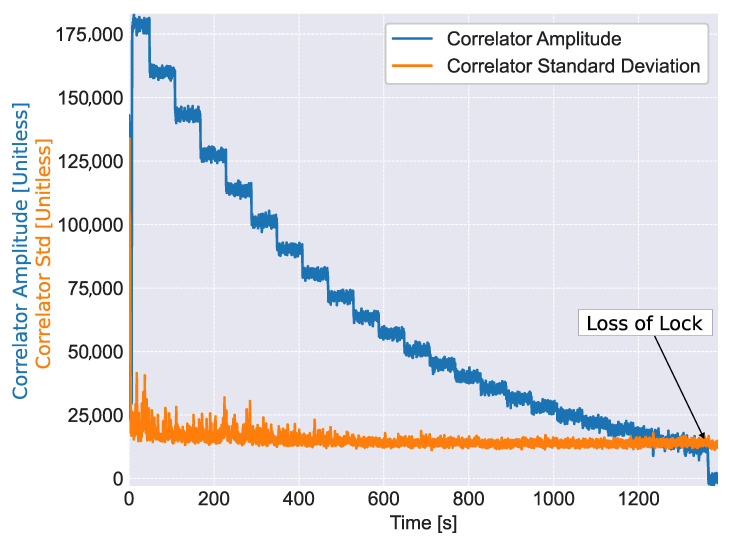
Amplitudes and standard deviations of the pilot prompt correlator estimated as part of the computation process of the LNL correlator. A single non-coherent integration is considered.

The proper functioning of the receiver, when implementing the LNL, is also demonstrated by Figure 8 that shows the amplitudes and standard deviation of the pilot prompt correlator estimated using (26) and (27). These estimates are used for the computation of the LNL correlator. The standard deviation is provided instead of the variance in order to be able to represent it together with the signal amplitude using the same scale. From the figure, it emerges that the signal amplitude is properly estimated using (27). The attenuation steps considered in the experiment are correctly reflected in the estimates of *A*. As expected, the noise standard deviation is practically constant and independent from the signal amplitude. When loss of lock occurs, after about 1400 s from the start of the test, the signal amplitude is below the noise standard deviation. This indicates the fact that the receiver is able to track signals with a post-coherent SNR, $\frac{A^{2}}{\sigma^{2}}$, below 0 dB.

**Figure 9:**
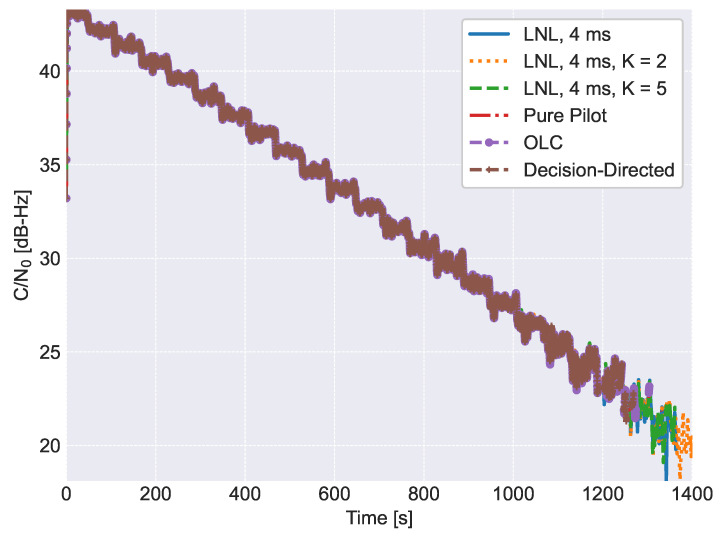
Comparison of the $C/N_{0}$ estimates obtained as function of time and for different processing configurations.

The $C/N_{0}$ obtained considering different combining techniques and different numbers of non-coherent integrations is shown in Figure 9. In all cases, the $C/N_{0}$ is estimated using only the pilot component. For this reason, all of the $C/N_{0}$ estimates coincide. In this case, the $C/N_{0}$ is used as a loss of lock indicator. PLLs implementing the LNL lose lock around 21 dB-Hz. While the best performance is obtained for the LNL correlator, for K=2, no significant improvements were expected in terms of loss of lock with respect to pure pilot processing, as analyzed in Section 5.2. Note that, loss of lock conditions also depends on the signal dynamics and update rate of the loop. This fact explains why, for K=5, no better loss of lock results are obtained.

**Figure 10:**
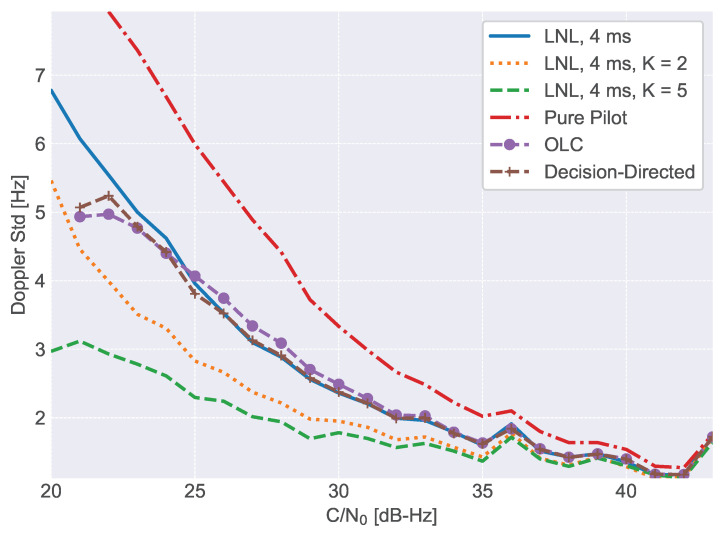
Standard deviation of the Doppler estimates obtained at the output of the tracking loop as a function of the $C/N_{0}$. Different processing configurations are considered.

Figure 9 also provides the $C/N_{0}$ values estimated for the OLC and decision-directed PLL: a slight degradation in terms of loss of lock is observed with respect to the other techniques. This is in agreement with the results discussed in Section 5.2 where the different techniques are compared using semi-analytic simulations. The real advantage of data/pilot combining techniques is with respect to the measurement generation process: less noisy measurements are obtained for the same $C/N_{0}$ conditions with respect to the pure pilot processing case. This fact was already studied in Section 5.1 and Section 5.2 considering the tracking jitter, that is the standard deviation of the carrier phase measurements. This fact is further studied in Figure 10, which shows the standard deviation of the Doppler frequency estimates obtained at the output of the tracking loop as a function of the $C/N_{0}$. Different numbers of non-coherent integrations, *K*, and different combining techniques are considered. The standard deviations have been estimated using the PLL filter outputs corresponding to the same $C/N_{0}$ values. The benefits of the LNL are clearly visible: less noisy Doppler frequency estimates are obtained with respect to the pilot-only case. The figure also reports the Doppler standard deviations for the OLC and decision-directed cases for a single non-coherent integration. The decision-directed PLL provides Doppler measurements with the same standard deviation as those generated by the LNL correlator for $C/N_{0}$ values greater than 25 dB-Hz. Small deviations occur for lower $C/N_{0}$ values until the decision-directed PLL loses lock. Also, the OLC is able to provide Doppler measurements with standard deviations similar to those obtained by the other combining approaches. These results are in agreement with the simulation findings discussed in Section 5.2.

The impact and benefits of non-coherent integrations for the LNL correlator are also evident from Figure 10.

## 6. Conclusions

In this paper, a combined data/pilot correlator is found using the ML approach. The correlator is made of a linear part, derived from the pilot component, and a non-linear one obtained from the data channel. A hyperbolic tangent term is used to remove data bits from the data correlator and opportunely weigh the data and pilot components. Since the data bits are removed through the multiplication by hyperbolic tangent terms, the integration time can be further extended in a non-coherent way. The correlator has been denoted as LNL given the two types of contributions from the data and pilot channel.

The LNL correlator has been analyzed under high and low signal power conditions: under weak signal conditions, the LNL correlator generalizes the squaring correlator used to extend the integration time on data channels. Under high power conditions the LNL becomes a form of decision-directed correlator.

Theoretical results have been supported by semi-analytic simulations and tests involving an SDR receiver. A good agreement between theoretical and simulation results has been found. Moreover, the advantages of the LNL correlator have been clearly demonstrated.

The LNL correlator has been compared with other combining strategies from the literature and in particular with the OLC and decision-directed PLL. The benefits and drawbacks of the different approaches have been studied: the advantages of the LNL correlator become more evident when a large number of non-coherent integrations are used.

The LNL approach combines data and pilot components into a single correlator, which has the same properties as a standard pilot correlator. In particular, it is not affected by residual bit transitions. For this reason, it can be directly used in algorithms currently using only the pilot correlator. This is the case, for instance, of several GNSS meta-signal tracking algorithms, which use pilot correlators only. Future work will investigate advanced meta-signal tracking algorithms using the LNL correlator and combining all components from the different frequencies forming the meta-signal.

## Figures and Tables

**Table 1:** Parameters used for the semi-analytic simulations.

Parameter	Value
Sampling frequency, $f_{s}$	4 MHz
Simulation runs	$5\times 10^{5}$
Coherent integration time	4 ms
Loop bandwidth	5/10/15 Hz
Loop order	3
Data/pilot power ratio	1

**Table 2:** Parameters used for the collection of the I/Q data generated using the Spirent GSS9000 simulator.

Parameter	Value
Sampling frequency, $f_{s}$	12.5 MHz
No. of bits	8 bits
Sampling type	Complex, IQ
Local oscillator frequency	1575.42 MHz
Attenuation steps	1 dB/min

**Table 3:** Tracking parameters used for the processing of the Galileo E1 signals.

Parameter	Value
PLL order	3
PLL bandwidth	15 Hz
DLL order	2
DLL bandwidth	2 Hz
Coherent integration time	4 ms

## Appendix A. Derivation of the Linear-Non-Linear Correlator

In order to determine the ML estimator for the residual phase error, Δφ, in (4) and (5), it is first necessary to derive the associated likelihood. Let’s start considering a single pair of data and pilot correlators. The variable $d_{i}$ in (5) models the impact of the bits on the data component and can be described as a scaled Bernoulli random variable:

(A1) $$d∼B\left(0.5\right)=\left\{\begin{array}{cc} 1 & \;\mathrm{with prob}.0.5\\ -1 & \;\mathrm{with prob}.0.5 \end{array}\right..$$

Finally, $\eta_{p,i}$ and $\eta_{d,i}$ are two i.i.d. complex circularly symmetric Gaussian random variables:

(A2) $$\begin{array}{cc} \eta_{p} & ∼N_{c}\left(0,\sigma^{2}I\right)\\ \eta_{d} & ∼N_{c}\left(0,\sigma^{2}I\right) \end{array}$$

where *I* is the 2×2 identity matrix.

Using these hypotheses, it is possible to derive the probability density functions (pdfs) of the pilot and data components for a single time epoch, *i*:

(A3) $$f\left(P_{p,i}\right)=\frac{1}{2\pi \sigma^{2}}\exp\left\{-\frac{1}{2\sigma^{2}}{\left|P_{p,i}-AR_{p}\left(\Delta \tau \right)e^{j\Delta \varphi }\right|}^{2}\right\}$$

(A4) $$f\left(P_{d,i}|d_{i}\right)=\frac{1}{2\pi \sigma^{2}}\exp\left\{-\frac{1}{2\sigma^{2}}{\left|P_{d,i}-kAd_{i}R_{d}\left(\Delta \tau \right)e^{j\Delta \varphi }\right|}^{2}\right\}.$$

Equation (A5) is conditioned with respect to the knowledge of the data bit, $d_{i}$. Using (A1), it is possible to find the overall pdf of the data component:

(A5) $$\begin{array}{cc} f(P_{d,i}) & =\frac{1}{4\pi \sigma^{2}}\exp\left\{-\frac{1}{2\sigma^{2}}{\left|P_{d,i}-kAR_{d}\left(\Delta \tau \right)e^{j\Delta \varphi }\right|}^{2}\right\}\\ \; & +\frac{1}{4\pi \sigma^{2}}\exp\left\{-\frac{1}{2\sigma^{2}}{\left|P_{d,i}+kAR_{d}\left(\Delta \tau \right)e^{j\Delta \varphi }\right|}^{2}\right\}\\ \; & =\frac{1}{2\pi \sigma^{2}}\exp\left\{-\frac{1}{2\sigma^{2}}\left[|P_{d,i}{|}^{2}+{\left|kAR_{d}\left(\Delta \tau \right)\right|}^{2}\right]\right\}\cosh\left\{\frac{A|k|R_{d}\left(\Delta \tau \right)}{\sigma^{2}}ℜ\left\{P_{d,i}e^{-j\Delta \varphi -j\varphi_{d}}\right\}\right\}. \end{array}$$

The phase $\varphi_{d}=∠k$ is known and used to align in phase the data and pilot correlators.

Given the independence of the data and pilot components, the joint pdf is obtained as the product of (A3) and (A5):

(A6) $$\begin{array}{cc} f(P_{p,i},P_{d,i}) & =\frac{1}{4\pi^{2}\sigma^{4}}\exp\left\{-\frac{1}{2\sigma^{2}}\left[{\left|P_{p,i}-AR_{p}\left(\Delta \tau \right)e^{j\Delta \varphi }\right|}^{2}+|P_{d,i}{|}^{2}+{\left|kAR_{d}\left(\Delta \tau \right)\right|}^{2}\right]\right\}\\ \; & \cdot \cosh\left\{\frac{A|k|R_{d}\left(\Delta \tau \right)}{\sigma^{2}}ℜ\left\{P_{d,i}e^{-j\Delta \varphi -j\varphi_{d}}\right\}\right\}. \end{array}$$

The log-likelihood function is obtained by taking the logarithm of (A6). After removing constant terms, the following likelihood is found:

(A7) $$L\left(\Delta \varphi \right)=\frac{AR_{p}\left(\Delta \tau \right)}{\sigma^{2}}ℜ\left\{P_{p,i}e^{-j\Delta \varphi }\right\}+\log\cosh\left\{\frac{AR_{d}\left(\Delta \tau \right)}{\sigma^{2}}ℜ\left\{{\tilde{P}}_{d,i}e^{-j\Delta \varphi }\right\}\right\}$$

where

$${\tilde{P}}_{d,i}=P_{d,i}k^{*}$$

is the data correlator corrected by *k*. In this way, ${\tilde{P}}_{d,i}$ is phase aligned with the pilot correlator and weighted with respect to the relative input data/pilot power.

Since we are concerned with the estimation of Δφ only, Δτ=0 is assumed in the following. Moreover, it is assumed that the correlator amplitude, *A*, and variance, $\sigma^{2}$, are known. In the implementation discussed in the paper, *A* and $\sigma^{2}$, are estimated.

In order to determine the ML estimator of Δφ, it is necessary to compute the derivative of (A7):

(A8) $$\begin{array}{cc} \frac{\partial L(\Delta \varphi )}{\partial \Delta \varphi } & =\frac{A}{\sigma^{2}}ℑ\left\{P_{p,i}e^{-j\Delta \varphi }\right\}+\frac{A}{\sigma^{2}}\tanh\left(\frac{A}{\sigma^{2}}ℜ\left\{{\tilde{P}}_{d,i}e^{-j\Delta \varphi }\right\}\right)ℑ\left\{{\tilde{P}}_{d,i}e^{-j\Delta \varphi }\right\}\\ \; & =\frac{A}{\sigma^{2}}ℑ\left\{\left[P_{p,i}+\tanh\left(\frac{A}{\sigma^{2}}ℜ\left\{{\tilde{P}}_{d,i}e^{-j\Delta \varphi }\right\}\right){\tilde{P}}_{d,i}\right]e^{-j\Delta \varphi }\right\}. \end{array}$$

Derivative (A8) is zero when the phase of the term between square brackets is equal to Δφ, that is when the term inside the imaginary part operator is real. This condition leads to the following equation

(A9) $$\Delta \hat{\varphi }=∠\left[P_{p,i}+\tanh\left(\frac{A}{\sigma^{2}}ℜ\left\{{\tilde{P}}_{d,i}e^{-j\Delta \hat{\varphi }}\right\}\right){\tilde{P}}_{d,i},\right]$$

where the $\hat{\cdot }$ has been added to denote that the solution of (A9) is an estimate of Δφ.

When a set of *K* data and pilot correlators are available, i.e., the integration time is extended beyond the coherent integration time, a similar equation is found:

(A10) $$\Delta \hat{\varphi }=∠\sum_{i=0}^{K-1}\left[P_{p,i}+\tanh\left(\frac{A}{\sigma^{2}}ℜ\left\{{\tilde{P}}_{d,i}e^{-j\Delta \hat{\varphi }}\right\}\right){\tilde{P}}_{d,i}\right],$$

which proofs Equation (6).
